# Medical Libraries in Hospitals

**Published:** 1906-02-17

**Authors:** Grace Whiting Myers

**Affiliations:** Assistant Librarian, Treadwell Library, Massachusetts General Hospital


### HOSPITAL ADMINISTRATION.

### CONSTRUCTION AND ECONOMICS.

### MEDICAL LIBRARIES IN HOSPITALS.

By Grace Whiting Myers, Assistant Librarian, Treadwell Library, Massachusetts General Hospital.

I venture to assert, at the risk of some opposition from fellow-librarians, that a medical library in a hospital is a necessity, and this is true even in a city which can boast of so beautiful and efficient a building as the one at Boston, Mass. The hospital library is unique, and in a measure stands by itself, answering to certain calls which the general library seldom, if ever, meets. Like the doctor, it must be ready for emergencies. It welcomes alike the physician and the surgeon, the house officer and the student, and supplies to each according to his needs. It is a room for study and for rest, for meetings and for consultations, and with sufficient material at hand to settle discussions and difficulties without delay. It is a quiet place in the midst of much activity, where the doctor may come and make the most of a few moments snatched at odd times during busy hours. It is a library situated in close association with hospital wards; and with the wealth of clinical history contained in hospital records at hand, theory and fact are brought into near relation, and an atmosphere created of study and investigation which to the doctor has a value beyond estimate. For, as the much-honoured Dr. Osler has expressed it: "To study the phenomena of disease without books is to sail an uncharted sea, while to study books without patients is not to go to sea at all."

#### The Need for Hospital Libraries.

As yet, the hospitals are few which contain real working libraries. Perhaps this is accounted for in several ways. The need may not be appreciated by the officers of the institution, space is not always available, and financial support cannot be furnished. The hospital is primarily a place for the care of the sick, and is so designed and built, with attention given to minute detail in point of administration, and with thoughtful care for the skilful and efficient treatment of patients. But the quiet library as a place of preparation for work is an essential worthy of consideration; for those who give freely time and thought and skill need a place for study, and need it in the hospital. The institution is never too young to make a beginning at a collection of books, and in its beginning the expense need not be large, for there will be those among the staff who, for the sake of personal convenience, if from no other motive, will be glad to donate from their own libraries and to send their own journals. Small sums of money appropriated by the trustees, or sent (as they sometimes are) in grateful acknowledgment of benefits received cannot be more wisely spent than in placing the little library upon a firm foundation.

#### A Case in Point.

As an example of the growth of such a library, and the point of usefulness to which it can attain, perhaps I cannot do better than give a brief sketch of the Treadwell Library of the Massachusetts General Hospital, which I have the honour to represent. It had its beginning in 1847, when a small sum was appropriated by the trustees of the hospital for the purchase of medical books. This appropriation was continued for several years, until in 1858 a bequest was made by Dr. John G. Treadwell, of Salem, Massachusetts, consisting of his large and valuable library, together with a fund of $5,000. With this donation the library now contained about 2,500 volumes. The first librarian, appointed in 1859, was Dr. Benjamin S. Shaw, who was then resident physician of the hospital. Later, a library committee was chosen from the members of the hospital staff, the chairman of the committee serving as librarian, until in 1897 it was thought advisable to have a regularly appointed assistant, whose time should be divided between the care of the books and the care of hospital records. At this time the number of books had increased to 4,872, and 28 periodicals were regularly received. Nothing had been done towards making a collection of reprints. In January 1900 there were 5,251 volumes, 38 periodicals, and 720 reprints. In January 1905 there were 6,037 volumes, 59 periodicals, and 2,172 reprints. These figures show that while the library has now been in existence for 58 years, one-third of its growth has been attained in the last eight years. Financial support is derived from the income of the fund of $5,000, already referred to, and from an annual assessment of the members of the Medical Board of the hospital, the hospital staff paying $5 each, and the out-patient staff $2 each. This buys new books, pays for periodical subscriptions, and for the binding of periodicals. Gifts of books are constantly received from our readers, and we have several donations of valuable journals.

One of the best hospital libraries is at the Johns Hopkins, in Baltimore, where during the school year there is an average daily attendance of one hundred. There is also one at the Boston City Hospital, containing something over 3,000 volumes, and one of about the same size at the McLean Hospital, Waverley; while others are fast proving their value in institutions in various parts of the country. In a list of 45 foreign medical libraries, recently furnished me, there are six located in hospitals, four of these being in England, one in Scotland, and one in far-away Japan.

#### What the Library should Contain.

It is worth while to consider just what a fully-equipped hospital library should contain. Of primary importance are the periodicals; they should be carefully chosen, and should be of the best in English, French, and German. They must cover the ground of general medicine and surgery, internal medicine, orthopædics, bacteriology, dermatology, neurology, and as many other "ologies" as the work of the hospital may demand. There should be standard works on anatomy with some good folios of plates, also standard editions of physiology, good up-to-date systems of medicine and of surgery, works on operative surgery, and on general pathology, year-books of various sorts, and, as far as means will permit, the best of all the new books. In addition, a collection of biographies, and some of the old classics for ever valuable in their lessons of humanity and conservatism. The ordinary text-books seem to be of comparatively little value; their day is short, they grow old in infancy (long before they are "sixty"), and are then consigned to what someone has aptly called the "library graveyard," wherein this epitaph might appropriately be inscribed:

Here lie books of ancient lore
Which once taught doctors to restore
To health the sick and lame.
Look kindly as you pass them by,
For words *you* write may sometimes lie
In silence just the same.

### A File of Official Materials.

Transactions of societies are always valuable, and medical and surgical reports of hospitals, also annual reports of hospitals, State Board of Health reports, and Government documents such as are issued from the Surgeon-General's Office, the Public Health and Marine-Hospital Service, and certain publications of the Department of Agriculture, and of the Bureau of Animal Industry. Then there are municipal documents which always command an interest in their respective localities. Nearly all of these reports may be had for the asking. Add now the invaluable index-catalogue of the Surgeon-General's Library, and the Index Medicus. Current issues of the former will be sent free by the Government to any library requesting them, and back volumes, if not out of print, may be obtained at small expense. The Index Medicus costs but $5 per year now, whereas it used to cost $25. There must be dictionaries, English, French, German, Italian, and Russian; a good general encyclopædia, and a gazetteer of the world. Some of these last-mentioned books may have a very un-medical sound, but there will be calls for every one.

### Management Means Success.

A word about management. All material, to be of practical use, must be classified, systematically arranged, and catalogued. The simplest plans are the best, for they are most easily understood by the general reader. Make all things available, and let the catalogue be plain enough to be useful. Rules there must be; for despised though they are, they mean protection. Let an effort be made to collect all pamphlets or reprints of articles written by members of the hospital staff, carefully cataloguing the same; and where reprints are not to be had, articles may be catalogued from the journal or volume in which they appear. Members of the Medical Board of the Massachusetts General Hospital have been asked to assist in this work by sending all such matter to the library, and they have willingly responded, in many instances expressing their appreciation that the writings of individual members were being gathered together. Preparation must be made for "emergency calls," when all the latest literature upon a given subject will be wanted immediately. In this connection bulletin work is of the greatest value. It not only familiarises the librarian with many subjects, and the names of writers upon such subjects, but it enables him to offer at a moment's notice a complete reference list of the latest literature upon a given topic. This work has been carried on in the Treadwell Library for about five years, the subjects chosen being either at the request of readers, or selected by the librarian on account of their current interest. There are about 90 such lists at present in this library. Some, which had but a passing interest, have not been continued, while others form an up-to-date index medicus, for references are added week by week. The librarian must needs be one who not only can handle the ordinary daily routine work, but he must be patient, tactful, the possessor of a good memory, keenly alert for every new thing, and he must take an individual interest in readers: in short, he must love his work. To him alone belongs the success or non-success of the library, and his field of work, though perhaps covering little space, is large and full of opportunity.

### Libraries and Nurses.

In closing may I speak one word for the nurses? Necessity really demands a good reference library for them, for while in the training school they are in a measure students of all branches of medicine, and must learn to meet the demands of advancing science, that they may furnish intelligent assistance to physician and surgeon. Here text-books for individual use are needed, but are secondary to reference books. An anatomy, a physiology, a good practice of medicine, a text-book of surgery, a volume of pediatrics, works upon dietetics, nursing, and kindred subjects, and a reliable medical dictionary should form the nucleus of this collection. Fifty or seventy-five well-chosen books will suffice; medical periodicals are not essential, but, of course, furnish valuable aid. According to statistics in an article recently published there are about 85 such libraries now in existence, the number of volumes in each varying from 25 to 200. Some of these were established, and are maintained, by the nurses themselves. Surely this shows that a need exists. Can it not be supplied?

